# DKK3 attenuates JNK and AP-1 induced inflammation via Kremen-1 and DVL-1 in mice following intracerebral hemorrhage

**DOI:** 10.1186/s12974-020-01794-5

**Published:** 2020-04-24

**Authors:** Yang Xu, Derek Nowrangi, Hui Liang, Tian Wang, Lingyan Yu, Tai Lu, Zhengyang Lu, John H. Zhang, Benyan Luo, Jiping Tang

**Affiliations:** 1 Key Laboratory of Non-coding RNA Transformation Research of Anhui Higher Education Institutes, Wannan Medical College, Wuhu, 241000 Anhui China; 2 Department of Basic Sciences, Division of Physiology, Loma Linda University School of Medicine, 11041 Campus St, Risley Hall, Room 219, Loma Linda, CA 92350 USA; 3 Department of Neurology, Wannan Medical College First Affiliated Hospital, Wuhu, 241000 Anhui China; 4 Department of Neurology, The First Affiliated Hospital, School of Medicine, Zhejiang University, Qingchun Road 79, Zhejiang, 310003 Hangzhou China; 5 Department of Anesthesiology, Loma Linda University School of Medicine, Loma Linda, CA 92350 USA

**Keywords:** Intracerebral hemorrhage, Inflammation, Dickkopf 3, Kremen-1, Dishevelled-1

## Abstract

**Background:**

Intracerebral hemorrhage (ICH) is the most devastating stroke subtype, with a poor prognosis and few proven treatments. Neuroinflammation is associated with ICH-induced brain injury and unfavorable outcomes. There is growing evidence that Dickkopf (DKK) 3 plays a key role in the adaptive anti-inflammatory and neuroprotective responses following intracerebral hemorrhage. This study aimed to evaluate the protective effects of DKK3 against brain edema and neuroinflammation in a mice model of ICH.

**Methods:**

Male, adult CD1 mice were subjected to sham or ICH surgery using a collagenase injection model. ICH animals received either recombinant DKK3, Kremen-1 siRNA, or DVL-1 siRNA. The neurobehavioral deficits were evaluated at 24 h, 72 h, and 28 days after ICH induction. Western blot and immunofluorescence were employed to examine the expression and localization of DKK3, Kremen-1, Dishevelled-1 (DVL-1), c-JUN N-terminal kinase (JNK), Activator protein-1 (AP-1), cleaved caspase-1, NF-κB, and IL-1β in the brain.

**Results:**

The expression of endogenous DKK3 and DVL-1 was transiently decreased after ICH compared to that in the sham group. Compared to the mice of ICH, exogenous rDKK3 administration reduced the brain water content and affected the neurological functions in ICH mice. Moreover, DKK3 was colocalized with Kremen-1 in microglia. Using a Kremen-1 or DVL-1 siRNA-induced in vivo knockdown approach, we demonstrated that the effects of DKK3 against ICH were mediated, at least partly, by the Kremen-1 and DVL-1 pathways.

**Conclusions:**

DKK3 improves the neurological outcomes, potentially by decreasing JNK/AP-1-mediated inflammation, thereby ameliorating the short- and long-term sequelae after ICH.

## Background

Intracerebral hemorrhage (ICH) accounts for 10–15% of all strokes that has a mortality that far exceeds those of ischemic strokes. The lack of specific therapeutic targets for ICH stresses the need for developing new therapeutic regimens [1]. The primary brain injury occurs within a few minutes to a few hours after the onset of ICH in which the initial hematoma causes local tissue destruction from the mass effect. A secondary injury phase occurs from the breakdown of cell debris and blood components which can persist in the area surrounding the adjacent hematoma for several weeks [2]. Increasing evidence has shown that neuroinflammation plays a critical role in the secondary brain injury following ICH [3–7].

Some studies have shown that Dickkopf (DKK) 3 promotes cell survival by suppressing superoxide-producing enzyme or by inhibiting inflammation [8, 9]. DKK3 is classified as a DKK glycoprotein family member that regulates cell fate during embryogenesis. DKK3 mediates potent antitumor effects, including reducing cell proliferation, anchorage-independent growth, and metastasis [10–12]. DKK3 antagonizes Wnt signaling by interacting with low-density lipoprotein receptor-related protein (LRP) 5/6 [13]. Therefore, DKK3 has been identified as a negative regulator of Wnt signaling in a complex and context-dependent manner [14]. Wnt principally involves the noncanonical (β-catenin-independent) and the canonical (β-catenin-dependent) signaling pathways [15]. Kremen-1 is a novel transmembrane receptor which function is Wnt inhibitory by removing LRP5/6 from the cell surface via clathrin-mediated endocytosis [16, 17]. Dishevelled-1 (DVL-1) is a central component protein that relays Wnt signaling in both canonical and non-canonical pathways whose activity and stability are strictly controlled [18]. The noncanonical pathways are also involved in c-JUN N-terminal kinase (JNK) activation, which produces dominant-negative JNK and prevents pressure overload. Additionally, calmodulin-dependent protein kinase II (CAMKII) phosphorylation was associated with increased histone deacetylase phosphorylation [18]. The activation of the JNK/Activator protein-1 (AP-1) signaling pathways is attributable to the increased expression of inflammatory factors in the brain [19].

DKK3, a secretory glycoprotein, is released from tissues of mice under physiological conditions in the retina, oral submucous, and brain [20–22]. In myocardial infarction conditions, the absence of DKK3 increased the mortality and infarct size, along with an increase in cardiomyocyte apoptosis and inflammation [9]. As a smooth muscle cell differentiation factor, the absence of DKK3 also leads to atherosclerotic plaque formation by increasing vascular inflammation [23]. The inhibition of inflammation in myocardial infarction might be associated with the regulation of the NF-κB pathway, which is also a cardioprotective regulator of pathological cardiac hypertrophy. This function largely occurs through the inactivation of apoptosis signal-regulating kinase (ASK1)/JNK/p38 signaling [24]. Consistent with above reports, DKK3 overexpression substantially alleviated cardiac hypertrophy and fibrosis [25, 26].

Currently, the mechanisms by which DKK3 modifies the inflammatory process in brain tissues are unclear. This study aims to determine the anti-inflammatory outcomes and mechanism of DKK3 in a mice model of ICH.

## Material and methods

### Animals

This study was approved by the Institutional Animal Care and Use Committee at Loma Linda University. All procedures were carried out in compliance with the guidelines for Animal Experimentation of Loma Linda University. A total of 174 male CD1 mice (8-week-old, weight 30–35 g; Charles River, Wilmington, MA, USA) were used. The animals were housed in a temperature-controlled environment with a 12-h light/dark cycle.

### Experimental protocol

**Fig. 1:**
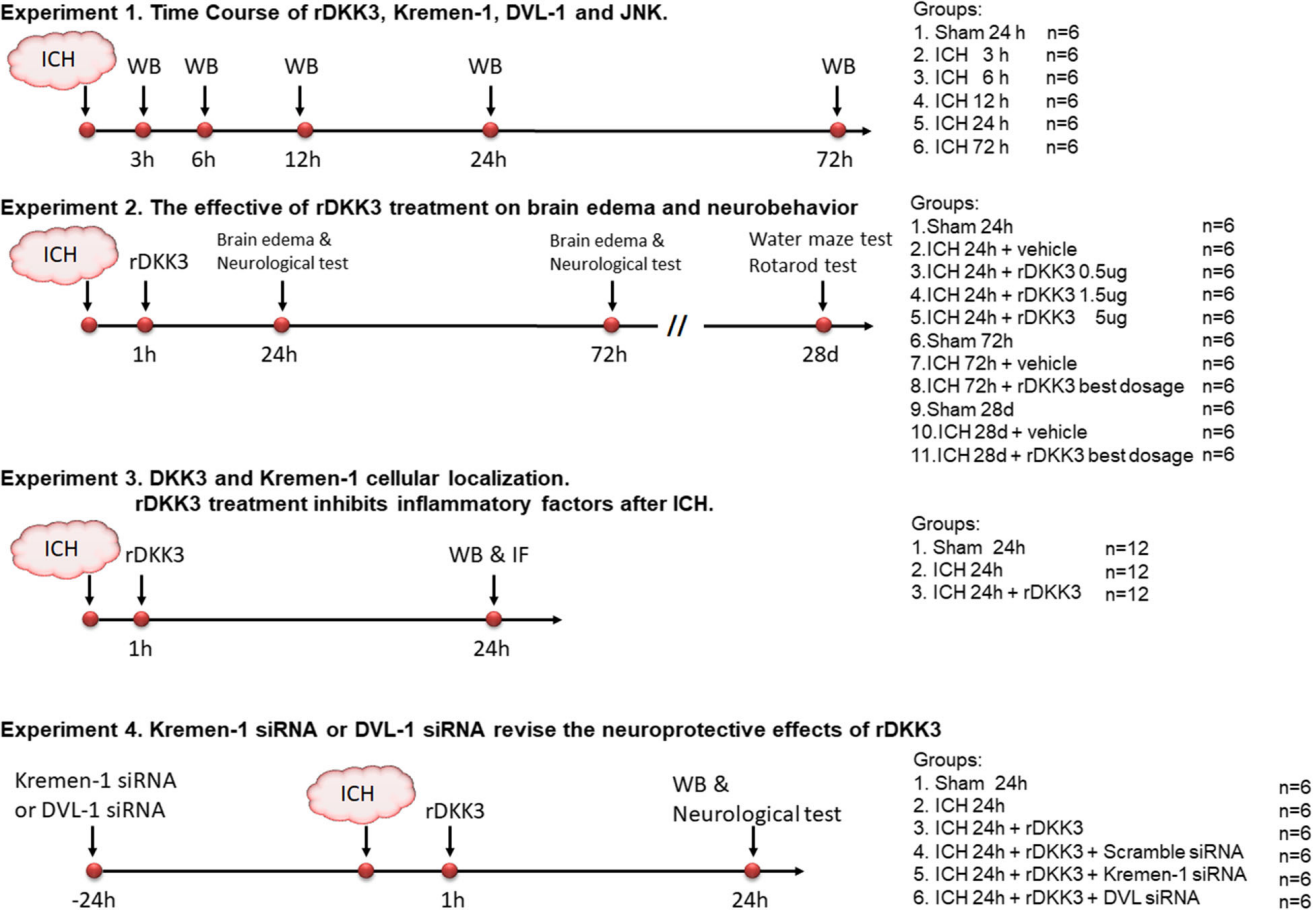
Study design. Representative figure showing experimental design and number of animals for each group. ICH, intracerebral hemorrhage; WB, western blot; rDKK3, recombinant DKK3

In the present study, all mice were randomly assigned to the following four separate experiments which are shown in the timeline of experimental design. The experimental groups and number of animals used in experiments are listed in Fig. 1.

#### Experiment 1

To evaluate the time course expression of endogenous DKK3 Kremen-1, DVL-1 and phosphorylated-JNK (p-JNK) in ICH mice, 36 animals were randomly divided into 6 groups for western blot: sham and 3, 6, 12, 24, and 72 h after ICH.

#### Experiment 2

To assess the short- and long-term neurobehavioral function, 48 mice were divided into 8 groups: sham, ICH 24 h, ICH 24 h + rDKK3 0.5 μg, ICH 24 h + rDKK3 1.5 μg, ICH 24 h + rDKK3 5 μg, sham 72 h, ICH 72 h, and ICH 72 h + rDKK3 best dosage. The brain edema and neurobehavior tests were evaluated at 24 and 72 h post-ICH. The Morris water maze test and rotarod test were conducted on day 28 post-ICH.

#### Experiment 3

To determine the DKK3 intracellular localization in ICH mice, 36 animals were randomly divided into 3 groups: sham, ICH, and ICH + rDKK3. The cellular localization of DKK3 was evaluated using double-labeling immunofluorescence labeling to co-localize DKK3 with ionized calcium-binding adaptor molecule 1 (Iba-1), neuronal-specific nuclear protein (Neun), and Kremen-1.

#### Experiment 4

To explore the underlying mechanisms of DKK3-mediated anti-inflammatory effects in ICH mice, Kremen-1 siRNA and DVL-1 siRNA were administered by intracerebroventricular (ICV) injection at 24 h before ICH induction. Thirty-six mice were randomly divided into 6 groups: sham, ICH, ICH + rDKK3, ICH + rDKK3 + scramble siRNA, ICH + rDKK3 + Krenam-1 siRNA, and ICH + rDKK3 + DVL siRNA. Neurobehavioral tests and western blot analysis were performed at 24 h after ICH.

### Intracerebral hemorrhage induction

ICH was induced via injection of collagenase into right basal ganglia as described [27]. After induction of anesthesia with ketamine (100 mg/kg) and xylazine (10 mg/kg), animals were positioned prone in a stereotaxic frame (David Kopf Instruments, Tujunga, CA, USA). An electronic thermostat-controlled warming blanket was used to maintain the core temperature at 37 °C ± 0.5 °C. The collagenase (0.075 units in 0.5 μl saline, VII-S; Sigma, MO, USA) was injected into the right basal ganglia. A 26-G needle was inserted with stereotaxic guidance coordinates 0.2 mm anterior, 3.5 mm ventral, and 2.2 mm lateral to the bregma at a rate of 0.1667 μl/min by a micro-infusion pump (Harvard Apparatus Inc., South Natick, MA, USA). Following the infusion, the needle was left in position for an additional 10 min after injection to prevent the leakage of collagenase, slowly retracted, and the incision was sutured. The sham group operation received only needle insertion.

### Assessment of neurological deficits

Neurological functions including modified Garcia test, wire hanging, beam balance, and limb placement were evaluated in a blinded fashion [28]. An independent researcher was blinded to the experimental design and conducted the exams at 24 h, 72 h, and 28 days after ICH. The Garcia test includes the evaluation of spontaneous activity, axial sensation, vibrissae proprioception, symmetry of limb movement, lateral turning, forelimb walking, and climbing.

In the long-term neurological study, we utilized the Morris water maze to test spatial learning capacity and memory function recovery according to previous studies [3, 27]. Starting from a semi-random location, each mouse is permitted to search for a partially submerged platform for 60 s. Next, the mice were guided to the platform and allowed to remain for 5 s. The probe trial was carried out on the last day of the exam. Following learning trial, the platform was removed. The swimming path, frequency of platform crossings, latency of first platform crossing, and frequency of correct quadrant crossings were recorded with a camera and linked to a computer tracking system (Noldus Ethovision, WA, USA). We also utilized the rotarod test to assess sensorimotor coordination and balance [29, 30]. The speed of a cylinder was slowly increased from 4 to 40 rpm over 5 min. The mice were allowed to run on the cylinders until they fell off and the times recorded. Each mouse was then tested three times, and the average retention time on rotarod was recorded. The mice were trained prior to testing with the average time elapsed from the rotation of the cylinder recorded as the baseline latency.

### Evaluation of brain water content

Mice were euthanized at 24 h or 72 h post-ICH. The brains were immediately removed and dissected into 5 parts: ipsilateral and contralateral basal ganglia and cortex. The cerebellum was used as the internal control [31]. Each part was weighed on an electronic analytical balance (APX-60, Denver Instrument, NY, USA) giving the wet weight (WW) and then dried at 100 °C for 24 h to determine the dry weight (DW). The brain water content was calculated as [(WW − DW)/WW] × 100%.

### Drug and siRNA administration

Three different formats of Kremen-1 siRNA or DVL-1 siRNA (OriGene Technologies, MD, USA) were diluted with transfection reagent (EntranserTM, Engreen Biosystem). ICV administration was performed at 24 h before ICH as described [32–34]. The Kremen-1 siRNA, DVL-1 siRNA, and scramble siRNA mixture (100 pmol in 2 μl) was delivered into the ipsilateral ventricle, administration at a rate of 0.667 μl/min. rDKK3 (SRP6268, Sigma-Aldrich, MO, USA) was dissolved in 10 μl of saline, and three different doses (0.5 μg, 1.5 μg, and 5.0 μg per mouse) were designed. rDKK3 was administered via intranasal route at 1 h post-ICH.

### Western blot analysis

Mice hemispheres were isolated and stored at − 80 °C until protein extraction. The ipsilateral brain hemispheres were homogenized in RIPA lysis buffer (sc-24948, Santa Cruz, TX, USA) and then centrifuged (14,000*g* at 4 °C for 30 min). Equal amounts of protein (50 μg) were loaded and subjected to electrophoresis on an SDS-PAGE gel. After being transferred to a nitrocellulose membrane, they are blocked with 5% nonfat milk (Bio-Rad Laboratories, Irvine, CA, USA). The membrane was incubated with the primary antibody overnight at 4 °C. The primary antibodies were used as follows: anti-DKK3 (1:1000, ab186409, Abcam, MA, USA), anti-Kremen-1 (1:500, ab86636, Abcam, MA, USA), anti-DVL-1 (1:1000, ab174679, Abcam, MA, USA), anti-AP-1 (1:200, NBP1-89544, Novusbio, CO, USA), anti-caspase-1 (1:1000, NBP1-45433, Novusbio, CO, USA), anti-IL-1β (1:500, sc-7884, Santa Cruz, TX, USA), and anti-p-c-Jun N-terminal kinase (p-JNK) (1:500, ab131499, Abcam, MA, USA). The blot bands were quantified using ImageJ (NIH). The results were expressed as ratio of the target band intensity to the band intensity of β-actin (1:1000, sc-58673, Santa Cruz, TX, USA) and then normalized to the mean sham group ratio.

### Immunofluorescence staining

Mice were perfused under deep anesthesia with isoflurane, followed by infusion of 4% paraformaldehyde. The brains were then removed and fixed in formalin at 4 °C overnight followed by dehydration with 30% sucrose in PBS. The frozen coronal slices (10 mm thick) were sectioned in cryostat (CM3050S; Leica Microsystems, Bannockburn). Brain slices were hydrated and blocked with 5% normal goat serum. Sections were incubated overnight at 4 °C with the following primary antibodies: anti-DKK3 (1:100, ab186409, Abcam, MA, USA) and anti-Kremen-1 (1:100, ab86636, Abcam, MA, USA). Then, they were incubated by appropriate fluorescence-conjugated secondary antibodies (1:100, AB2337972, AB2338059, AB2340432, or AB233887, Jackson ImmunoResearch Laboratories, PA, USA) at room temperature for 2 h. Sections were observed using an OLYMPUS BX51 microscope.

### Statistical analysis

All values are presented in the text as mean ± standard deviation (SD). Western blot data were analyzed using one-way ANOVA with Tukey post hoc tests. Behavior data were analyzed using one-way ANOVA on ranks with Tukey post hoc tests or repeated measures ANOVA when appropriate. All histological data were analyzed using one-way ANOVA with Student-Newman’s post hoc tests. Statistical significance implies *p* < 0.05.

## Results

### Endogenous levels of DKK3, Kremen-1, DVL-1, and p-JNK in ICH mice

**Fig. 2:**
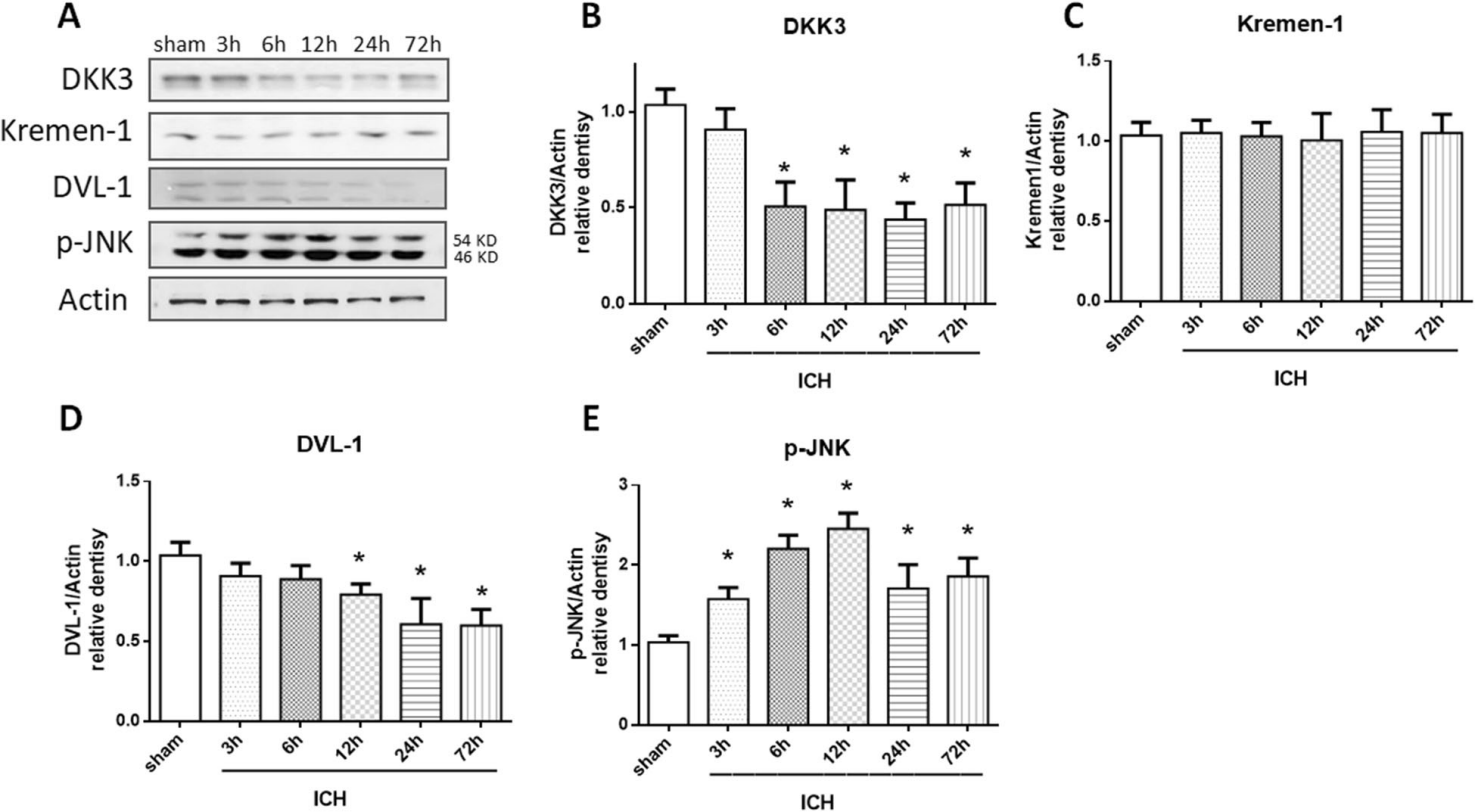
Expression of DKK3, Kremen-1, DVL-1, and p-JNK in the ipsilateral hemispheres after intracerebral hemorrhage. Representative western blotting images of DKK3, Kremen-1, DVL-1, and p-JNK (**a**). Bar graphs of the quantitative analysis of DKK3 (**b**), Kremen-1 (**c**), DVL-1 (**d**), and p-JNK (**e**) expression in the ipsilateral hemisphere after ICH. Data are expressed as the mean ± SD, *p < 0.05 versus sham, *n* = 6 animals per group

Western blotting showed that the expression of DKK3 transiently decreased after ICH (*p < 0.05 versus sham; Fig. 2b) compared to that of the sham group at 6 to 72 h after ICH. Kremen-1 showed no change at any time point (Fig. 2c). DVL-1 decreased at 12 h after ICH (*p* < 0.05 versus sham; Fig. 2d). An elevation of p-JNK was observed at 3 h and reached a peak at 12 h after ICH. The level of p-JNK began to decline at 24 h but remained statistically significant to sham through 72 h (*p* < 0.05 versus sham; Fig. 2e).

### rDKK3 improved the neurological functions and reduced the brain water content at 24 and 72 h after ICH

**Fig. 3:**
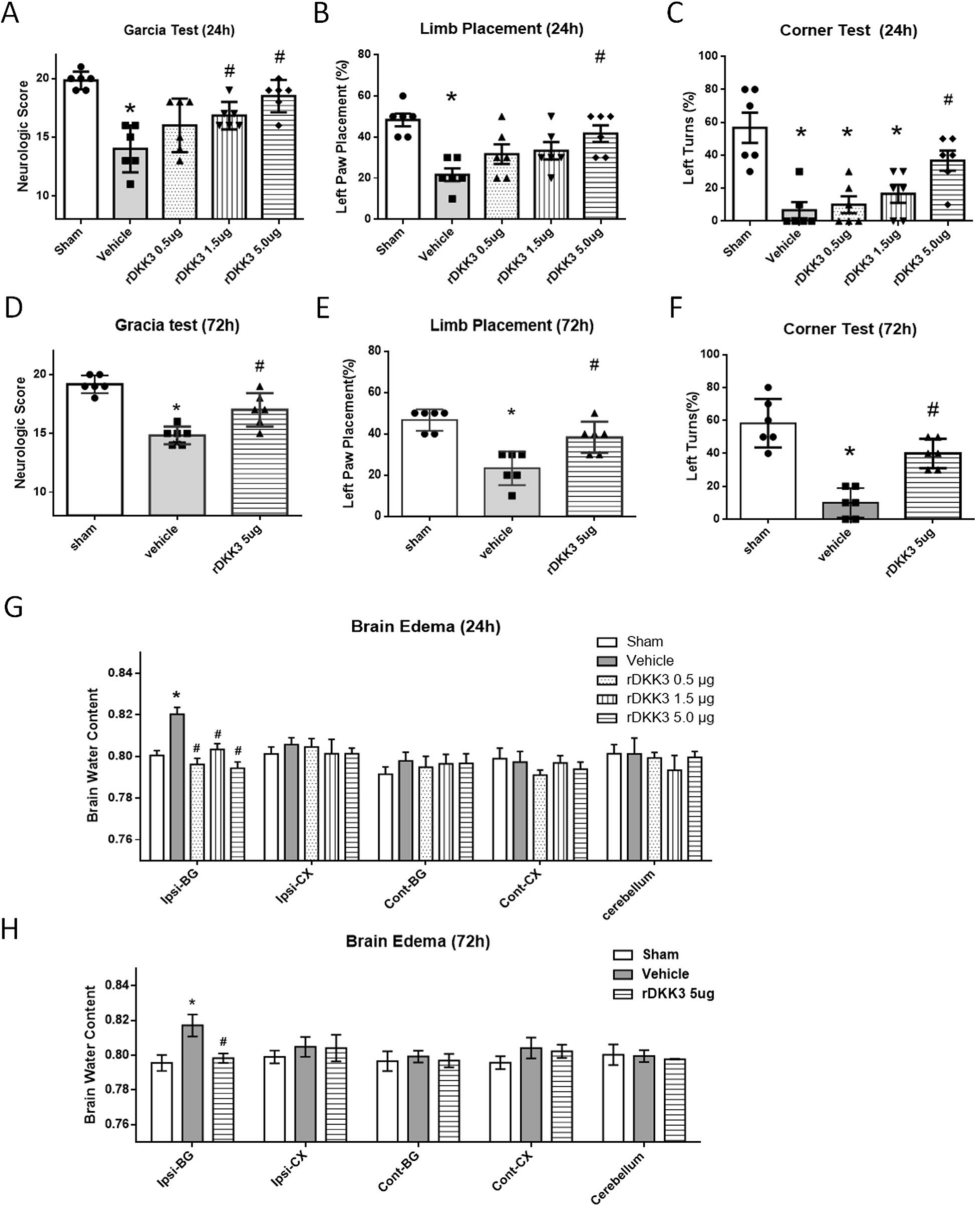
Effects of rDKK3 on the neurological outcome, brain water content, escape latency, and swimming distance after ICH. Garcia test score (**a**), left forelimb placement (**b**), and corner turn test (**c**) at 24 h after ICH. Garcia test score (**d**), left forelimb placement (**e**), and corner turn test (**f**) at 72 h after ICH. Brain water content 24 h (**g**) and 72 h (**h**) after ICH. Data are expressed as the mean ± SD, *p < 0.05 versus sham; ^#^*p* < 0.05 versus vehicle, *n* = 6 animals per group

Three different doses of rDKK3 (0.5 μg, 1.5 μg, and 5.0 μg) were administered intranasally at 1 h after ICH induction. The mice in the vehicle group showed statistically decreased performance in the Garcia test, limb placement test, and corner turn test at both 24 h and 72 h after ICH compared to those of the sham group (Fig. 3a–f). rDKK3 (5.0 μg) improved the neurological functions in all the same tests at 24 and 72 h after ICH compared to those in the vehicle group (*p* < 0.05 versus vehicle).

The brain water content in the vehicle group showed significant elevation in ipsilateral basal ganglia at both 24 and 72 h (*p < 0.05 versus sham; Fig. 3g, h). Compared to the vehicle group, rDKK3 treatment reduced the ICH-induced brain water content in the ipsilateral basal ganglia at 24 and 72 h after ICH (^#^*p* < 0.05 versus vehicle). The 24-h results indicated that the high dose of rDKK3 (5.0 μg) showed the greatest improvements and was thus selected as the dosage for the 72-h experiment and other experiments.

### rDKK3 improved long-term neurobehavior

**Fig. 4:**
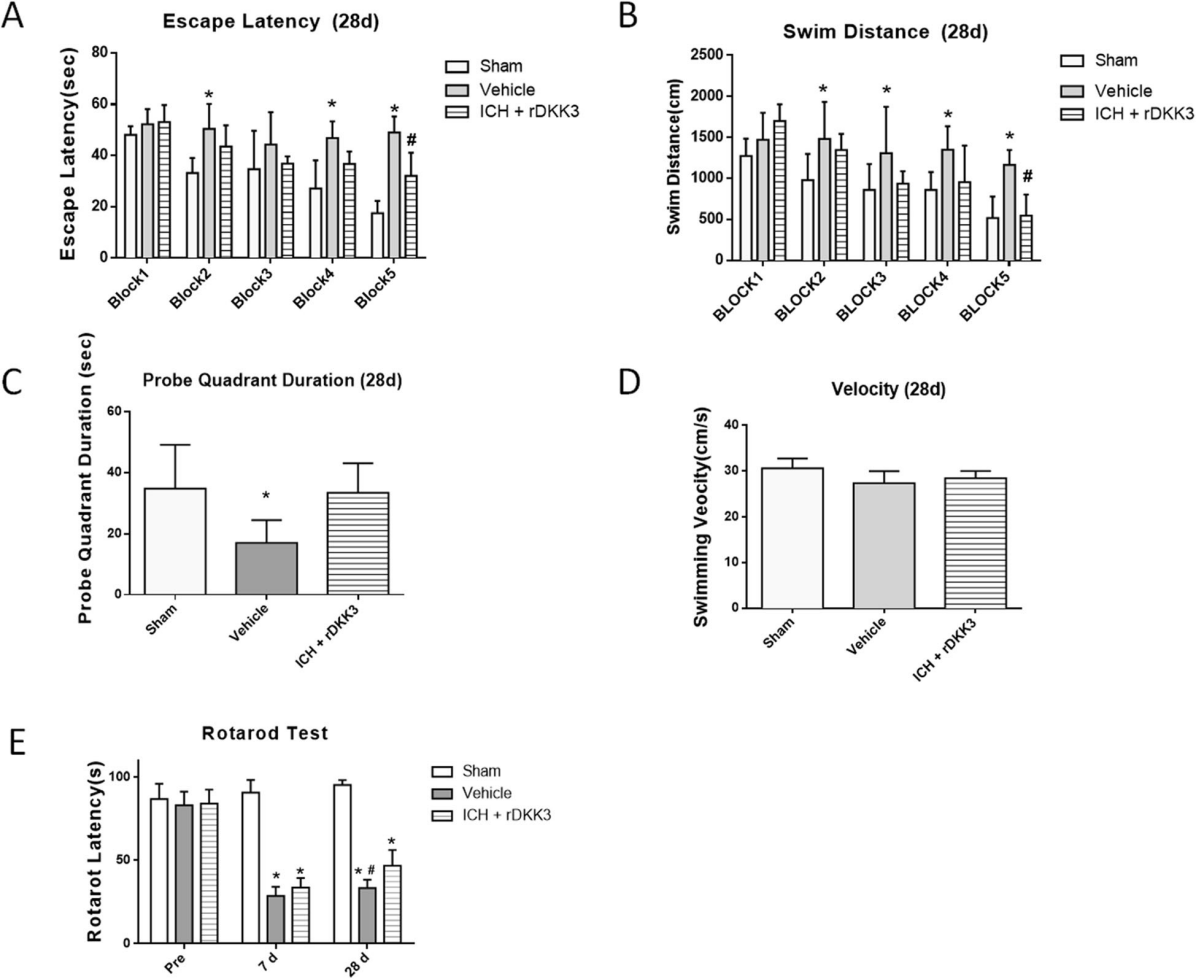
Administration of rDKK3 improved neurological function at 28 days after ICH. Morris water maze test of spatial learning performance was analyzed with the escape latency (**a**) and swimming distance (**b**). The duration in the probe quadrant (**c**) and velocity (**d**) 28 days after ICH. Rotarod test 7 days and 28 days after ICH (**e**). *p < 0.05 versus sham; ^#^*p* < 0.05 versus vehicle, *n* = 6 animals per group

To examine the long-term neurological impairment and neurological function, the Morris water maze test and rotarod test were performed at 4 weeks post-ICH [35]. The results showed that the escape latency and swimming distance of the vehicle group were significantly longer compared to sham (*p < 0.05 versus sham; Fig. 4a, b). The rDKK3 group exhibited an improved performance in escape latency and swimming distance on the 5th day of training compared to vehicle. Though not statistically significant to vehicle (*p* > 0.05), rDKK3 did show improvement by not being statistically significant to sham in the probe quadrant duration (Fig. 4c). However, comparing the velocity did not show significant changes between any of the groups (*p* > 0.05; Fig. 4d). The rotarod test showed no changes between the vehicle and rDKK3 groups at 7 days after the surgery. However, rDKK improved the performance in the rotarod test at 28 days after ICH (^#^*p* < 0.05 versus vehicle; Fig. 4e).

### rDKK3 treatment reduces the expression of inflammatory markers after ICH

**Fig. 5:**
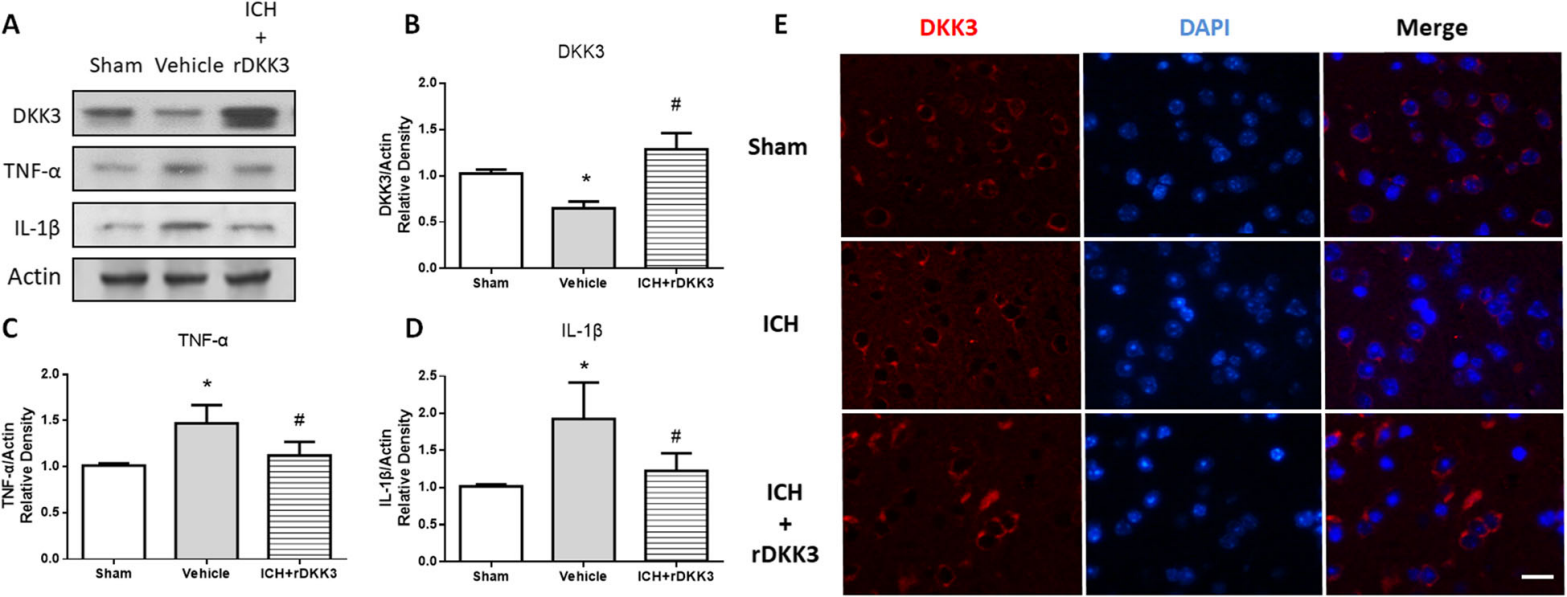
Expression of DKK3, TNF-α, and IL-1β. Representative western blotting images of DKK3, TNF-α, and IL-1β in the ipsilateral brain tissue (**a**). Bar graphs of the quantitative analysis of DKK3 (**b**), TNF-α (**c**), and IL-1β (**d**) expression after ICH or rDKK3 treatment. Data are expressed as the mean ± SD, *p < 0.05 versus sham; ^#^*p* < 0.05 versus vehicle, *n* = 6 animals per group. Representative images of DKK3-positive cells in the perihematomal brain tissue (**e**). Scale bar: 50 μm, *n* = 6 animals per group

The vehicle group showed a decreased expression of DKK3 after ICH at 24 h consistent with our results (*p < 0.05 versus sham; Fig. 5a, b). After rDKK3 administration, the total level of DKK3 increased when compared with the vehicle group (^#^*p* < 0.05 versus vehicle; Fig. 5a, b). Inflammatory factors, including TNF-α and IL-1β expression, increased after ICH in the vehicle group compared to sham (*p < 0.05; Fig. 5a, c, d). rDKK3 administration reversed these changes and showed significantly decreased expression of TNF-α and IL-1β as compared to vehicle (^#^*p* < 0.05; Fig. 5a, c, d). The immunofluorescence results showed that the fluorescence intensity of DKK3 outside of the cell membrane in vehicle and DKK3 treatment groups was slightly reduced when compared to sham (Fig. 5e).

### Co-localization of DKK3 and Kremen-1 in microglia and neurons after ICH

**Fig. 6:**
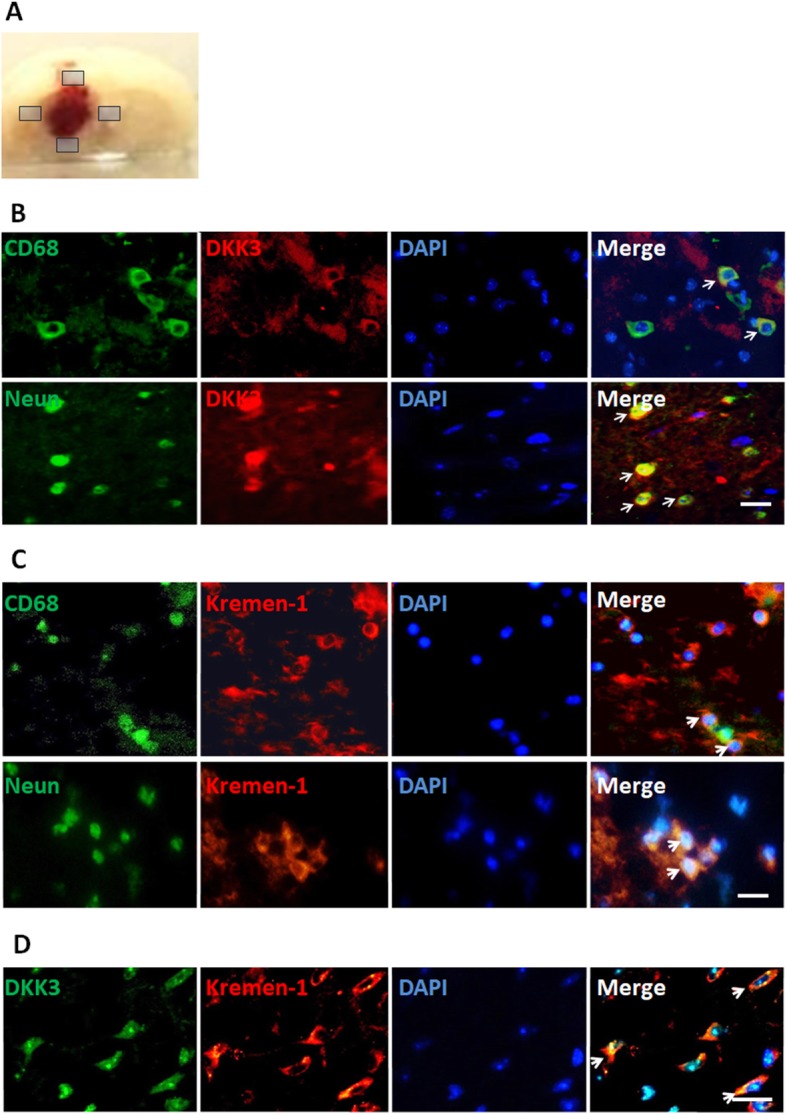
Endogenous expression of DKK3 and Kremen-1 after ICH in microglia and neurons. Brain samples were obtained from the perihematomal area of the brain tissue (shown with a gray shadow) 24 h following ICH (**a**). Representative images of immunofluorescence staining showing the expression of (**b**) DKK3 (red) and (**c**) Kremen-1 (red), colocalizing with the activated microglia marker CD68 (green) and neuronal marker Neun (green). DKK3 (green) colabeled with Kremen-1 (red) (**d**). Scale bar: 20 μm, *n* = 6 animals per group

Dual-immunofluorescence staining was performed in the perihematomal brain tissue 24 h following hemorrhage induction. The staining showed that both DKK3 and Kremen-1 were colocalized with microglia (CD68) and neurons (Neun; Fig. 6b, c). Interestingly, most of Kremen-1 was visualized as a ring. Immunofluorescence co-labeling of DKK3 with Kremen-1 was detected in the vehicle group (Fig. 6d).

### Knockdown of Kremen-1 or DVL-1 reversed the effects of the rDKK3-mediated inhibition of inflammation after ICH

**Fig. 7:**
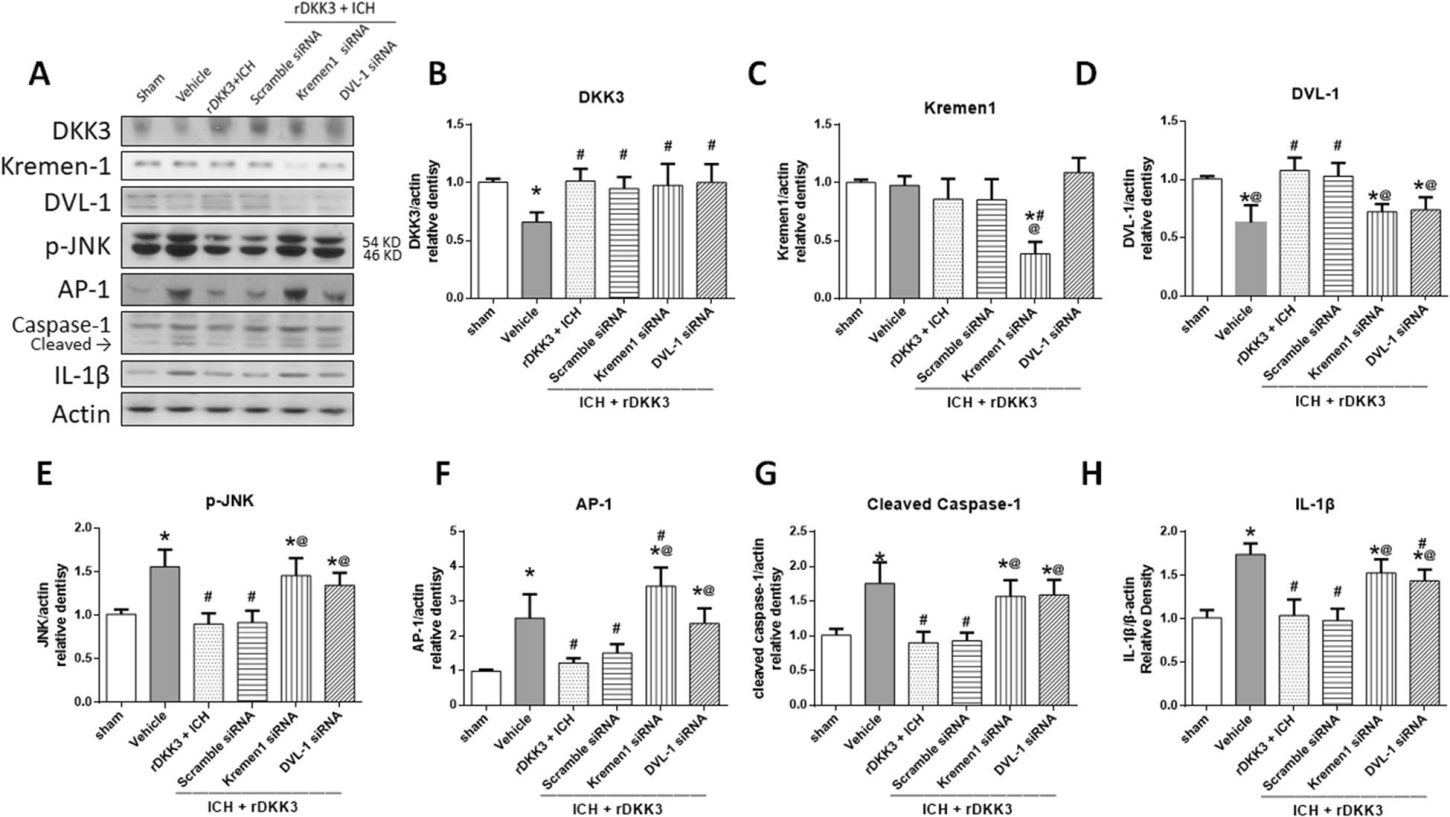
Western blot of rDKK3/Kremen-1/DVL-1 mechanistic pathway. Representative western blot bands (**a**) and the densitometric quantification of DKK3 (**b**), Kremen-1 (**c**), DVL-1 (**d**), p-JNK (**e**), AP-1 (F), cleaved caspase-1 (**g**), and IL-1β (**h**) in the sham, vehicle, rDKK3, rDKK3 + scrambled siRNA, rDKK3 + Kremen-1 siRNA, and rDKK3 + DVL-1 siRNA groups 24 h after ICH. Data are expressed as the mean ± SD. *p < 0.05 versus sham; ^#^*p* < 0.05 versus vehicle; ^@^*p* < 0.05 vs rDKK3 + scramble siRNA group, *n* = 6 animals per group

The ICH mice were given rDKK3 in addition to either Kremen-1 siRNA, DVL-1 siRNA, or scramble siRNA. Western blot analysis was done at 24 h after ICH (Fig. 7a). Results showed that the expression of DKK3 and DVL-1 was significantly decreased at 24 h after ICH (*p < 0.05 versus sham group; Fig. 7b, d). Additionally, p-JNK, AP-1, cleaved caspase-1, and IL-1β were significantly elevated in the vehicle group compared to the sham group (*p < 0.05; Fig. 7e–h). rDKK3 administration showed increased DKK3 and DVL-1 expression (^#^*p* < 0.05; Fig. 7b, d) and decreased p-JNK, AP-1, cleaved caspase-1, and IL-1β compared to vehicle (^#^*p* < 0.05; Fig. 7e–h). Kremin-1 siRNA and DVL-1 siRNA showed successful knockdown of expression of Kremin-1 and DVL-1 respectively (*p < 0.05; Fig. 7c, d). Kremin-1 siRNA and DVL-1 siRNA given with rDKK3 showed reversal of expression changes seen from rDKK3 treatments alone. Both Kremin-1 siRNA and DVL-1 siRNA groups showed increasing expression of inflammatory factors p-JNK, AP-1, cleaved caspase-1, and IL-1β compared to sham (*p < 0.05) and the scramble siRNA group (^@^*p* < 0.05; Fig. 7e–h). Additionally, Kremin-1 siRNA showed a significant elevation of AP-1 versus vehicle (^#^*p* < 0.05; Fig. 7f), and DVL-1 siRNA showed increase in IL1β compared to vehicle (^#^*p* < 0.05; Fig. 7h).

### The rDKK3-mediated improvement in neurobehavior functions was reversed with Kremen-1 siRNA or DVL-1 siRNA

**Fig. 8:**
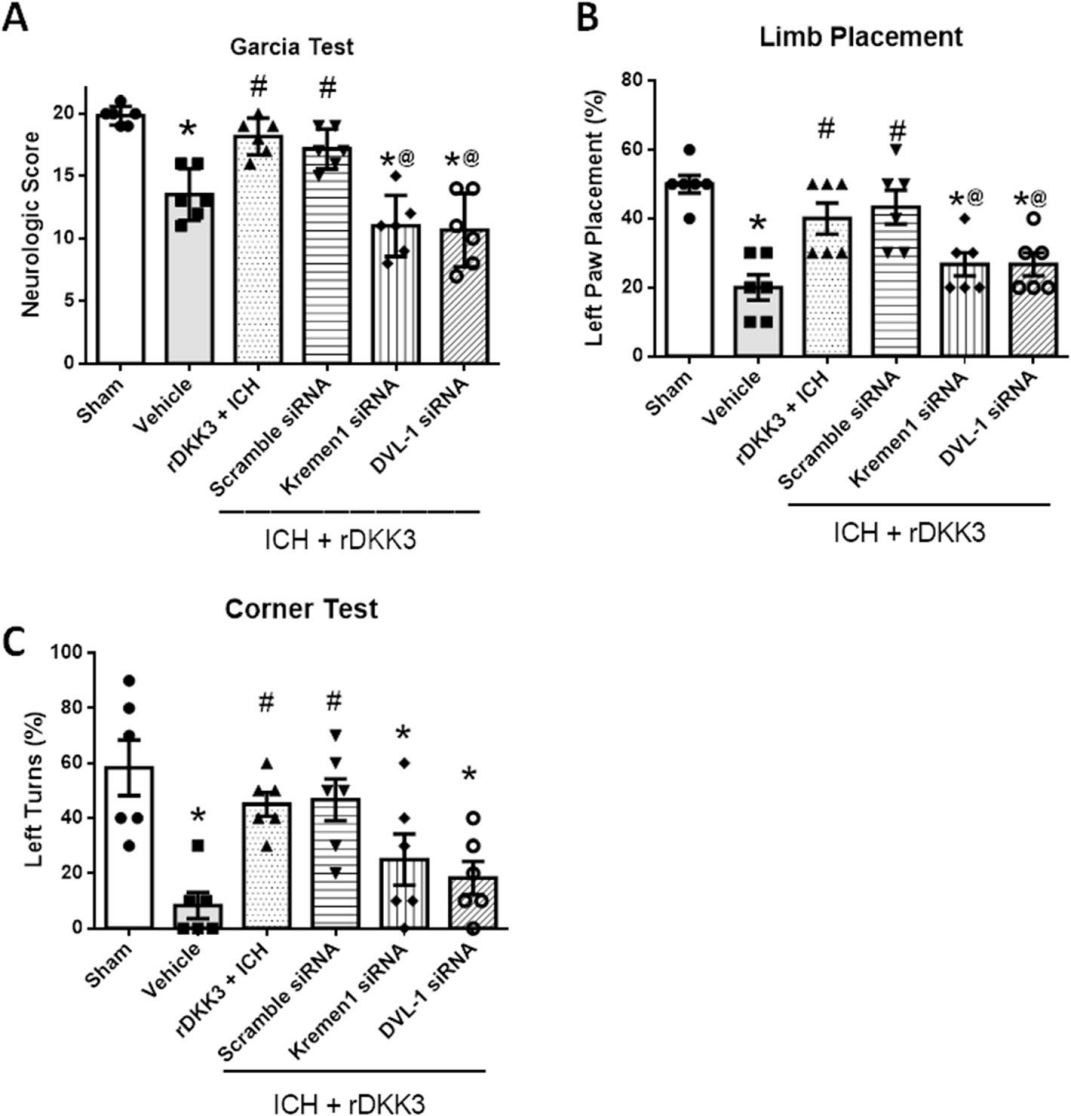
Neurological function at 24 h showing effects of Kremen-1 and DVL-1 siRNA. Modified Garcia test (**a**), limb placement test (**b**), and corner turn test (**c**) in the sham, vehicle, rDKK3, rDKK3 + Scrambled siRNA, rDKK3 + Kremen-1 siRNA, and rDKK3 + DVL-1 siRNA groups 24 h after ICH. Data are expressed as the mean ± SD. *p < 0.05 versus sham; ^#^*p* < 0.05 versus vehicle; ^@^*p* < 0.05 vs rDKK3 + scramble siRNA group, *n* = 6 animals per group

The results showed that the neurobehavior function significantly improved at 24 h after rDKK3 administration, as evaluated by the modified Garcia test, limb placement test, and corner turn test consistent with our results (^#^*p* < 0.05 versus vehicle; Fig. 8a–c). Kremin-1 siRNA and DVL-1 siRNA given with rDKK3 showed reversal of these improvements and showed a significant decrease in neurobehavior scores compared to sham (*p < 0.05; Fig. 8a–c).

## Discussion

In this study, we demonstrate that the administration of rDKK3 showed beneficial outcomes following the conditions of ICH. The inflammatory response in ICH is related to the recruitment of inflammatory cells and to the activation of a series of inflammatory cascades. A reduction in inflammatory infiltrates is associated with an attenuation of brain injury of ICH [36–38]. Previously, DKK3 was observed at high levels in the liver, heart, kidney, and brain [39–42]. DKK3 overexpression substantially alleviated cardiac hypertrophy and fibrosis [10]. We showed that rDKK3 inhibits ICH-induced inflammation, leading to improved neuroprotection after experimental ICH in mice.

Intranasal administration allows many drugs to avoid BBB and reach the cerebrospinal fluid via olfactory sensory neurons [43]. Thus, in the present study, rDKK3 was intranasally administered. Three doses of rDKK3 (0.5 μg, 1.5 μg, and 5.0 μg) were evaluated in the ICH mouse model. Our results showed that treatment with 5.0 μg of rDKK3 improved neurological function and reduced cerebral edema at 24 and 72 h after ICH (Fig. 3). Additionally, we found that long-term neurological deficits were also improved based on the water maze and rotarod experiments (Fig. 4). This suggests that DKK3 may be a neuroprotective therapy in a setting of ICH.

Although it has been shown that DKK3 protects neurons against a variety of toxic insults via mediating vascular endothelial growth factor (VEGF), the complete biological action of DKK3 is still not fully understood. Previous studies have also shown that DKK3 may reduce activation of inflammatory pathways such as JNK1/2 and p38 pathways as observed in myocardial infarction [9]. In this study, we found that a decreased DKK3 expression was accompanied by an increase of inflammatory factors after ICH. By treating ICH mice with rDKK3, we observed significant decreases in the release of inflammatory factors such as TNF-α, cleaved caspase-1, and IL-1β (Fig. 5). Microglia activation during brain injury and neuroinflammation has previously been linked to the secretion of pro-inflammatory cytokines including IL-1, IL-6, and TNF-α [44, 45]. In our experiment, we observed that rDKK3 administration decreased the expression of TNF-α and IL-1β, suggesting a reduction in microglial activation. These findings suggest that DKK3 may modulate the inflammatory response found after ICH (Figs. 5a, 7a).

The specific receptors and related signaling pathways that DKK3 interacts with are still controversial compared to those of the other DKK family members. It has been proposed that the DKK3-Kremen-1 interaction may affect Wnt signaling [46]. Nicola confirmed that Kremen-1, but not lipoprotein receptor-related protein 6 (LRP6), immunoprecipitated with DKK3 in cancer-associated fibroblasts, and the mRNA levels of Kremen-1 were not altered after DKK3 silencing [47]. In the present study, Kremen-1 did not show any change in expression during the same timeframe when DKK3 levels were observed to be reduced after ICH. Utilizing Kremin-1 siRNA together with rDKK3 reduced the protective ability shown with rDKK3 alone (Fig. 2). This suggests the connection of DKK3 to the Kremen-1 receptor.

We also found that DKK3 and Kremen-1 were expressed in neurons and microglia after ICH (Fig. 6). Of interest was our observation of the Kremen-1 pattern changes in neurons. Kremen-1 formed a ring at the edge of the cell membrane suggesting it is cell surface membrane bound when there was a reduction in DKK3 as seen during the ICH setting. This is consistent with other studies such as in a tumor microenvironment where DKK3 and Kremen-1 colocalized to internal structures; however, after DKK3 silencing, Kremen-1 localized to the cell periphery [47]. DKK3 distribution was also found to be disrupted following ICH injury showing decreased expression (Fig. 5e). These findings suggest that endogenous DKK3 shapes the responsiveness of neurons to inflammatory insults. Previous research has reported endogenous DKK3 decreases ischemic brain damage in mice and acts as a protective molecule in cultured neurons [22]. On human Alzheimer’s disease tissue, DKK3 is expressed in neurons and in blood vessel walls in the brain [48]. Consistent with our staining in Fig. 6b, DKK3 and neurons co-localize. DKK3 was also found which localized to glia in mouse retina [21, 49]. Our immunohistochemical analysis also showed that DKK3 expression was detected in CD68-positive cells. This possibly shows that DKK3 is produced by microglia and stimulates the same microglia to reduce its own activation and acts as a protective molecule. In support of the mechanism, DKK3 overexpression in the liver alleviated chronic inflammation, as evidenced by lower levels of pro-inflammatory cytokines and genes such as IL-1β, IL-6, TNF-α, MCP-1, and iNOS similar to what we observed in our study [39].

We also found that the ICH injury caused a gradual decline in the endogenous expression of DVL-1 from 12 to 72 h after ICH. The reduction in DKK3 and DVL-1 after ICH may be a protective mechanism of DKK3. Yu and colleagues also demonstrated that DVL-1 and JNK are potential signaling pathways that relay DKK3 signaling in endothelial cells [50]. However, to our knowledge, we are the first to demonstrate its signaling pathways being potentially associated in microglia. The loss of DKK3 led to the inflammatory cell infiltration accompanied by an increased p-JNK expression in the brain, supporting the deterioration of neurobehavior (Fig. 5).

Based on our results, we suggest that DVL-1 plays a major role in the downstream signaling involved in the DKK3 mechanism. These results show that rDKK3 administration increases DVL-1 and decreases JNK, AP-1, cleaved caspase-1, and IL-1β (Fig. 7). DVL-1 is composed of three conserved domains (N-terminal DIX domain, PDZ domain, and a C-terminal DEP domain). Among these, the PDZ domain plays an important role in protein interactions. This domain binds to the membrane-bound receptor and to other signal transduction molecules in the cytoplasm to distinguish between suitable binding partners [51]. Specifically, for the pathways we are exploring, the transmembrane receptor Kremen-1 may compete with the canonical signaling pathway for the interaction with DVL-1 and downstream molecules through its PDZ domain. However, this action of DVL-1 is not dependent on Wnt or its downstream effector β-catenin. We reduced their expression utilizing Kremen-1 siRNA and DVL-1 siRNA respectively. In the knockdown of either Kremen-1 or DVL-1, the rDKK3-mediated inhibition of inflammation after ICH is reduced. This suggests that DKK3 forms a ternary complex with Kremen-1 receptors thus allowing fine-tuning of downstream signaling. Furthermore, the neurobehavioral findings indicated that the neuroprotective effect of DKK3 after ICH is mediated by DVL-1 and Kremen-1 (Fig. 8).

Our data strongly suggest that the suppression the downstream protein JNK/AP-1 largely account for the neuroprotective action of DKK3. However, one of the major limitations of this study is that we paid special attention to the involvement of DKK3 to the Kremen-1 receptor and that other potential mechanisms have not been investigated. DKK3 has been reported to inhibit inflammation through the noncanonical Wnt signaling pathway in a model of atherosclerosis. This mechanism promoted reendothelialization and induced endothelial cell migration [50]. Furthermore, DKK3 has also been observed in a cardioprotective model to interrupt the ASK1–JNK/p38 signaling cascades [9]. In addition, though we primarily explored the protective effects of DKK3 on the inflammatory process, we also observed that neurons may be affected by other pathways as well potentially contributing to the improved behavioral outcomes we observed. Therefore, DKK3 might represent a promising therapeutic target for ICH and neuron injury as well.

## Conclusion

**Fig. 9:**
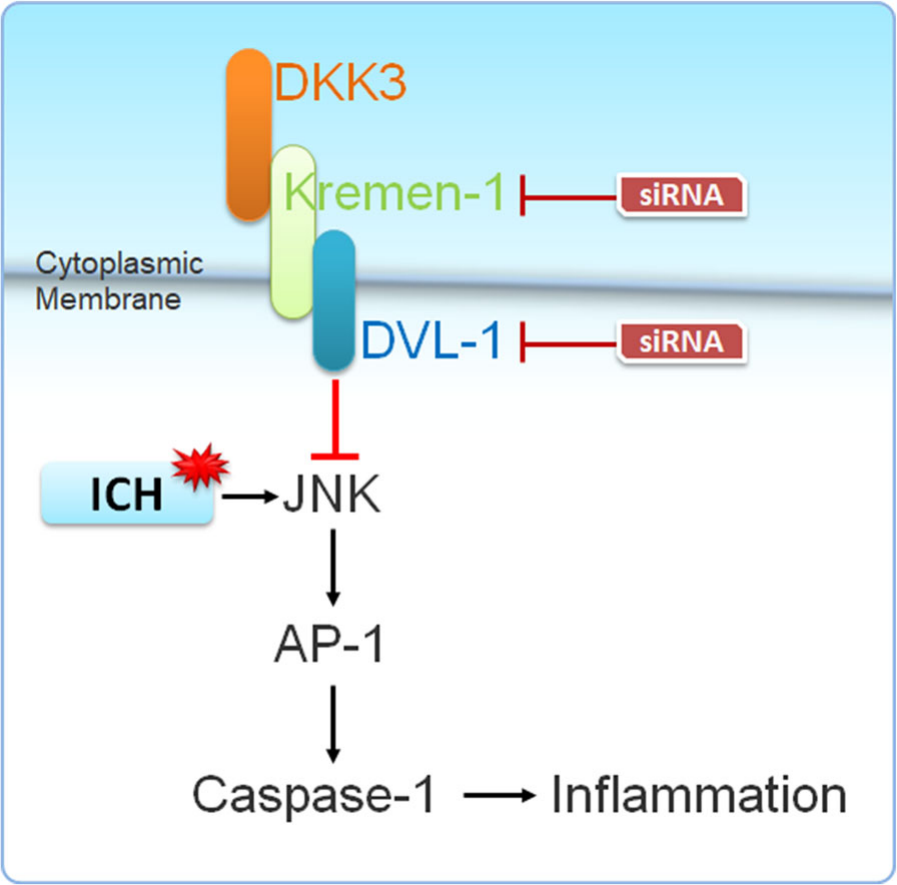
Mechanism representation in a microglia. ICH induces JNK/AP-1 activation thus leading to an increase in caspase-1 and producing an inflammatory response. Administration of rDKK3 activates Kremen-1 (phosphorylated) complex with DVL-1, thus reducing JNK/AP-1 signaling and reducing inflammation. Administration of Kremin-1 and DVL-1 siRNA should reduce the effects of DKK3/Kremen-1/DVL-1 thus reducing the beneficial effects of rDKK3 on ICH-induced inflammation

This study proposed that DKK3 inhibits the inflammatory reactions induced by ICH. The underlying mechanism of the protective role of DKK3 in ICH is through Kremen-1 and DVL-1 to inhibit JNK/AP-1 signaling pathway (Fig. 9). The mechanism of DKK3 signaling is complex and is dynamically regulated during brain development through its crosstalk with many signaling pathways. Further research is needed to elucidate the precise mechanism by which DKK3 regulates DVL-1 signaling. Our results indicate a neuroprotective role in reducing inflammation in an experimental model of ICH. Therefore, we propose that DKK3 administration may provide a promising therapy to the pathology of ICH and brain injury.

## Data Availability

The datasets analyzed during the current study are available from the corresponding author on reasonable request.

## Abbreviations

AP-1: Activator protein-1
Cont-BG: Contralateral basal ganglia
Cont-CX: Contralateral cortex
CAMKII: Calmodulin-dependent protein kinase II
DKK: Dickkopf
ICH: Intracerebral hemorrhage
IL-1β: Interleukin-1 beta
Ipsi-BG: Ipsilateral basal ganglia
Ipsi-CX: Ipsilateral cortex
JNK: c-JUN N-terminal kinase
LRP: Lipoprotein receptor-related protein
VEGF: Vascular endothelial growth factor
